# The relationship between obesity and body compositions with respect to the timing of puberty in Chongqing adolescents: a cross-sectional study

**DOI:** 10.1186/s12889-017-4681-1

**Published:** 2017-08-18

**Authors:** Fang He, Peiyu Guan, Qin Liu, Donna Crabtree, Linli Peng, Hong Wang

**Affiliations:** 1 School of Public Health and Management, Chongqing Medical University, Research Center for Medicine and Social Development, Collaborative Innovation Center of Social Risks Governance in Health, Chongqing Medical University, Chongqing, 400016 China; 2 Chongqing Health Information Center, Chongqing, 400016 China; 3 Duke Global Health Institute, Duke University, Durham, NC 27708 USA; 4 School of Medicine, Duke University Medical Center, Durham, NC 27705 USA

**Keywords:** Obesity, Body compositions, Timing of puberty, Adolescents

## Abstract

**Background:**

It is well known that excess adiposity during childhood may influence pubertal development. However, the extent to which body compositions vary in throughout puberty in boys and girls is currently unknown. The aim of this study was to investigate whether obesity and body compositions correlate with the timing of puberty in boys and girls.

**Methods:**

By random cluster sampling, our study analyzed data from 1472 students (690 girls, 782 boys) aged 6–17 years from two schools in the Chongqing area. Data were collected by physical examination of weight, height, and skinfold thicknesses. Testicular volume was measured in boys and breast development in girls. By which we got the indicators of obesity, timing of puberty and body compositions. Probit regression analysis was used to group subjects into early puberty (>P_25_), on-time puberty (P_25_ ~ P_75_), and delayed puberty (<P_75_). Chi-square test was applied to assess the distribution differences of fat levels among puberty groups by sex. Multivariate analysis of covariance was used to examine the association between timing of puberty and body composition indicators.

**Results:**

There were significant differences in the distribution of fat levels (normal weigh, overweight, obesity) among different pubertal groups in different sex (boys:*χ2* = 10.639, *P* = 0.031; girls:*χ2* = 63.232, *P* = 0.000). Multivariate analysis of covariance showed that percentage of body fat, fat mass and fat-free mass in the delayed puberty girls were significantly lower than the girls in the on-time puberty and early puberty, while body density had the opposite result (all *P* < 0.001). Body density, percentage of body fat, fat mass and fat-free mass of boys all had no significant difference among different timing of puberty groups (all *P* > 0.05).

**Conclusions:**

In girls, delayed puberty was negatively correlated with Obesity, percentage of body fat, fat mass and fat-free mass, and positively correlated with body density. But in boys, delayed puberty was only negatively correlated with Obesity, the relation between puberty and body compositions was not found.

## Background

Puberty represents a period during development of gradual transition from childhood to adulthood. The WHO Expert Committee states that adolescent ages range from 10 to 20 years old. However, there are significant differences in the age of onset, speed of development and age of maturity. Studies have found that the onset and development of puberty are primarily controlled by the interaction between genetic and environmental factors. At present, there is no consensus on the mechanism for initiation of puberty, but the more widely accepted view is that the central nervous system, particularly the hypothalamic–pituitary–gonadal (HPG) axis, plays a decisive role [1].

Early puberty is defined as the appearance of adrenocortical function, gonad function, and menarche earlier than the first quartile of the age distribution in children [2], and this phenomenon has received increasing attention during the past couple of decades. Some studies have found that girls who experience an earlier menarche age were taller in early adolescence but shorter in early adulthood than those who matured later, despite having a greater peak height velocity and greater post-menarche increase in height [3].

In recent decades, the prevalence of childhood and adolescent obesity has been rapidly increasing and has become a major public health concern both in China and worldwide. Studies have shown that obesity is associated with earlier puberty onset among adolescent girls and young female adults [4–7]. For instance, girls who had more body fat content were more likely to experience menarche at younger ages [8] and early maturing adolescent girls were almost twice as likely to be overweight than average-maturing girls [9]. In boys, however, some research has suggested that early-maturing boys were less likely to be obese compared to boys who matured at the average rate [4].

In the majority of studies, obesity is defined by Body Mass Index (BMI) level; however, this metric is not entirely suitable for children [10], and the sensitivity of using BMI to identify children with obesity was generally only low to moderate [11, 12]. As early as 400 BC, the Greeks put forward the concept of body composition [13]. Behnke et al. [14] divided body composition into fat mass (FM) and fat-free mass (FFM) and found that the proportion of body compositions between males and females is different. Burton RF [15] found that the relationships between FFM and FM were linear and FM were correlate with height. But the interrelationships among FM, FFM, total body mass (BM) and height have long been studied in relation to the BMI [16, 17]. Although BMI (kg/m ^2^) is widely used as a measure of adiposity, it is a measure of excess weight relative to height (overweight) rather than excess adiposity (obesity) [18]. A study of 2554 school children found a correlation of *r* = 0.58 between BMI and body fatness (assessed by skinfolds) among boys who were relatively fat, but no association (*r* = 0.01) among leaner boys [19]. Norgan NG et al. [20] found that the relationships between BMI and body compositions differed with age, sex, and ethnicity and body shape. Moreover, recent study reported that skinfold thickness during childhood and adolescence is a better predictor of high body fatness in adults than BMI [21]. Moreover, changes in skinfold thickness or in percentage body fat have been considered more precise than BMI to monitor obesity, thus providing greater confidence for epidemiological applications. Therefore, the assessment of obesity should ideally be based on measurement of body compositions [22].

This study assessed whether obesity correlates to timing of puberty, specifically determining whether higher skinfold thickness, percentage of body fat (%BF), FM and FFM associate with early puberty. More importantly, a comprehensive examination of the association in boys and girls between early puberty and obesity incidence provides useful insights to advance our understanding of the relationship between these phenomena.

## Methods

### Study sites and subjects

This survey was a cross-sectional study. A three-stage random cluster sampling method was used in this survey. In stage one, a central district was chosen in Chongqing. In stage two, two nine-year schools, with grades one to six belonging to primary school and grades seven to nine belonging to junior middle school, were chosen. One of the schools was located in an urban area, while the other was in a village. Students in these two schools represented both high and low levels of economic development. In the third stage of sampling, girls from grades two and three and boys from grades three and four were selected from each school; simultaneously, both girls and boys in grades seven and eight were selected. Three to five classes were selected from each grade. The grades this study chose to examine mostly encompass all the early and delayed individuals with respect to puberty development. Girls from grade two and boys from grade three were chosen due to the difference in onset of early puberty, being approximately seven years old in girls and about eight years old in boys.

A total sample of 1472 students (690 girls, 782 boys) aged 7 to 17 years were selected in this survey. All individuals completed a physical examination. The study was approved by the ethic committee of Chongqing Medical University. Written informed consent was also obtained from a parent or guardian on behalf of each child.

### Data collection

This study mainly based on a physical examination, including weight, height, tricep skinfold thickness, subscapular skinfold thickness and suprailiac skinfold thickness. At the same time, breast development in girls, and testis volume in boys, were examined. The evaluation methodology of these indicators was as follows:

#### Pubertal Assessment

Breast stages (stage 1–5) were assessed by visual inspection and palpation using the rating scales of Marshall and Tanner [23]. The volume of each testis was estimated by comparative palpation with an orchidometer. To classify boys puberty status, volume of testis (VOT) were divided into 4 stages: stageI,VOT < 4 ml; stageII, 4 ml ≤ VOT < 12 ml; stage III, 12 ml ≤ VOT < 20 ml; stage IV, VOT ≥ 20 ml. Median ages at breast stage 2 and 4 ml of VOT are considered the beginning of puberty for our use [24, 25]. Subjects were grouped using the quartiles of the decimal age adjusted for Tanner stages of puberty and gender. For example, in Tanner stage 1, children and adolescents were considered as ‘in early puberty’ if they were in the first quartile of the decimal age for that stage and gender. The same procedure was used for all the other Tanner stages. Those in the fourth quartile of the decimal age are considered as ‘those with delayed puberty’, and if they were between first and fourth quartile of the decimal age, they are considered to be ‘on-time’ [6]. It is worth noting that girls who were in breast 1 and boys whose VOT < 4 ml were regarded as ‘not having begun puberty’, and were not included in later analyses. The formula used to calculate the decimal age of P_25_, P_75_ for each stage are as follows: Probit (P) = α + Σßx.

#### Anthropometry

Measurements of weight, height and skinfold thickness were collected from all subjects. Standing height was measured to the nearest 0.1 cm using a height meter. Weight was measured on a digital electronic scale with a precision of 0.1 kg. The children were weighed without shoes, wearing light clothing. Skinfold thickness was taken with a Harpendencalliper on the right side of the body with a precision of 0.1 mm. We calculated BMI (= weight [kg]/height[m] ^2^) and the sum of skinfold thickness (SST) (= triceps skinfold thickness + subscapular skinfold thickness) for each individual as well. It is worth noting that the final values of skinfold thickness should be only half of the measured results, as we measured the double skin fold. All surveyors received standardized training before this survey.

#### Definition of Obesity and Overweight

To define obesity and overweight, children aged 7–18 years were categorized according to sex-age-specific BMI cut offs recommended by the Chinese Working Group on Obesity (WGOC) [26].

#### Body Compositions

Firstly, SST was used to calculate Body Density (BD). BD formulas are shown as follows [1]: BD = 1.0879–0.0015 × SST for both7-11 years boys and girls; BD = 1.0868–0.00133 × SST for 12-14 years boys, BD = 1.0888–0.00153 × SST for 12-14 years girls; BD = 1.0977–0.00146 × SST for 15-18 years boys, BD = 1.0931–0.0060*SST for 15-18yearsgirls. Next, we used the following formula to calculate %BF with BD: %BF = (4.57/BD-4.142) × 100. Finally, FM and FFM were used as additional measures of body composition according to Maynard et al. [10]: FM = weight × %BF and FFM = weight – FM.

### Data analysis

Using the probit regression analysis, we calculated the quartiles of the decimal age of each Tanner stage for girls’ breast and boys’ testis, by which subjects were grouped into early puberty (>P_25_), on-time puberty (P_25_ ~ P_75_), and delayed puberty (<P_75_). Chi-square test was used to explore whether there were differences between boys’ and girls’ timing of puberty, and compared the distribution difference of fat levels between pubertal groups by sex. Finally, BD, %BF, FM and FFM as the independent variable, pubertal groups (early puberty, on-time puberty and delayed puberty) as dependent variable, multivariate analysis of covariance were used to examine whether body compositions varied with respect to different pubertal groups. Data management and data analysis were performed with SPSS Version 19.0. Any result with a *P*-value less than 0.05 was considered statistically significant. When compared the difference in each obesity and body composition measure by 3 groups of puberty timing, Chi-square test and multivariate analysis of covariance sufficed with correction of *p*-values.

## Results

### Characteristics

Table 1 presents the characteristics of the study sample, including mean age, the number of different school and grade, anthropometric and body compositions grouped by sex.

**Table 1:** Characteristics of Study Participants

Indicators		Boys (*n* = 782)	Girls (*n* = 690)
Age (Mean ± SD)		11.72 ± 2.09	11.02 ± 2.66
School			
	Urban school	421	333
	Village school	361	357
Grade			
	Grade two	-	203
	Grade three	109	102
	Grade four	225	-
	Grade seven	221	181
	Grade eight	227	204
Anthropometric			
	Height (cm)	147.57 ± 13.84	142.37 ± 14.59
	Weight(kg)	41.81 ± 13.70	38.12 ± 13.72
	Triceps skinfold (mm)	6.60 ± 3.04	7.54 ± 3.29
	Subscapular skinfold (mm)	5.03 ± 3.22	5.86 ± 2.70
	Suprailiac skinfold (mm)	5.14 ± 3.28	7.72 ± 3.74
Body compositions			
	BD(kg/L)	1.07 ± 0.01	1.06 ± 0.01
	%BF	12.51 ± 3.35	15.43 ± 4.44
	FM(kg)	5.49 ± 3.12	5.66 ± 3.40
	FFM(kg)	36.47 ± 11.13	32.74 ± 11.07

*BD:* body density, *%BF:* percentage of body fat, *FM:* fat mass, *FFM:* fat-free mass, *BMI:* body mass index

As shown in Tables 2, 441 boys and 407 girls had entered puberty. By using the probit analysis to calculate the quartiles of the decimal age of each Tanner stage for girls’ breast and boys’ testis, the subjects were grouped into three groups: early puberty, on-time puberty, and delayed puberty. Over one eighth of the boys (12.7%) were classified as early puberty, nearly three quarters of boys (70.3%) were on-time puberty, and 17.0% were regarded as experiencing delayed puberty. Approximately one fifth of girls (19.7%) were experiencing early puberty, 38.0% of girls developed on-time, and 42.3% of girls were developing later than others. The percent of early and delayed puberty girls was significantly higher than boys (*P* = 0.007, *P* < 0.001, separetely), and the percent of on-time puberty girls was significantly lower than boys (*P* < 0.001).

**Table 2:** Chi-square test of timing of puberty between boys and girls

	Boys (*n* = 441)	Girls (*n* = 407)	*χ2*	*P*
Early puberty	56 (12.7%)	80 (19.7%)	7.609	0.007
On-time puberty	310 (70.3%)	155 (38%)	88.673	<0.001
Delayed puberty	75 (17.0%)	172 (42.3%)	65.389	<0.001

### Association between obesity, overweight and puberty timing

The prevalence of obesity and overweight in boys and girls in different pubertal groups were presented in Table 3. There were significant differences in the distribution of fat levels (normal weigh, overweight, obesity) among different pubertal groups in different sex (boys:*χ2* = 10.639, *P* = 0.031,girls:*χ2* = 63.232, *P* = 0.000).

**Table 3:** Singer factor analysis of fat level and timing of puberty by sex

Groups	Boys (*n* = 441)			Girls (*n* = 407)		
	Normal weight	Overweight	Obesity	Normal weight	Overweight	Obesity
Early puberty	41 (73.2%)	10 (17.9%)	5 (8.9%)	50 (62.5%)	15 (18.8%)	15 (18.8%)
On-time puberty	257 (83.2%)	30 (9.7%)	22 (7.1%)	131 (84.4%)	20 (13.0%)	4 (2.6%)
Delayed puberty	70 (93.3%)	2 (2.7%)	3 (4.0%)	166 (96.5%)	5 (2.9%)	1 (0.6%)
*χ2*		10.639			63.232	
*P*		0.031			0.000	

boys: “Early puberty” VS “On-time puberty”, *P* = 0.155; “On-time puberty” VS “Delayed puberty”, *P* = 0.000, “Early puberty” VS “Delayed puberty”, *P* = 0.000

girls: “Early puberty” VS “On-time puberty”, *P* = 0.000; “On-time puberty” VS “Delayed puberty”, *P* = 0.000, “Early puberty” VS “Delayed puberty”, *P* = 0.000

### Body compositions compared to timing of puberty

As shown in Table 4, BD, %BF, FM and FFM of boys had no significant difference among different timing of puberty group (all *P* > 0.05). But all indicators in the delayed puberty were significantly lower than in the on-time puberty and the early puberty girls, except for BD had the opposite result (all *P* < 0.017).

**Table 4:** Multivariate analysis of covariance of body compositions and timing of puberty (mean ± SD)

	Indicators	Timing of puberty			F	*P*
		Early puberty	On-time puberty	Delayed puberty		
Boys (*N* = 441)	BD(kg/L)	1.070 ± 0.010	1.071 ± 0.008	1.071 ± 0.009	0.513	0.599
	%BF	13.105 ± 3.958	12.665 ± 3.388	12.503 ± 3.454	0.516	0.597
	FM(kg)	6.526 ± 3.649	6.446 ± 3.233	6.136 ± 3.127	0.316	0.729
	FFM(kg)	40.851 ± 9.209	42.397 ± 8.109	41.115 ± 9.221	1.269	0.282
Girls (*n* = 407)	BD(kg/L)	1.059 ± 0.009	1.062 ± 0.012	1.067 ± 0.014^a, b^	12.199	0.000
	%BF	17.306 ± 3.804	16.160 ± 5.057	14.243 ± 5.859^a, b^	10.888	0.000
	FM(kg)	8.829 ± 4.099	7.861 ± 3.147	6.272 ± 2.831^a, b^	19.700	0.000
	FFM(kg)	41.553 ± 14.080	40.163 ± 6.826	37.302 ± 5.508^a, b^	8.684	0.000

^a^ “Early puberty” VS “Delayed puberty”, *P* < 0.017

^b^ “On-time puberty” VS “Delayed puberty”, *P* < 0.017

## Discussion

This study examined how children’s fat levels (obesity and overweight) and body compositions varied with respect to timing of puberty development based on the evaluation of breast development in girls and the volume of each testis in boys according to the Tanner stage standard.

Although the mechanism of association between obesity and early puberty in girls remains to be elucidated, the relationship between obesity and early puberty are consistent. Obesity and overweight are positively correlated with early puberty [4–7, 27, 28]. This study also found that there were significant differences in the distribution of overweight and obesity among the groups of early puberty, on-time puberty, and delayed puberty.

In boys, the relationship is still controversial. Wang [4] examined the influence of early sexual maturation on fat in 1520 US boys aged 8–14 years and found that early sexual maturation was negatively correlated with obesity. However, Lee et al. [29] reported the opposite in 705 American boys. In Chinese boys, the relationship seems to be positive [30]. In this study, the rates of overweight and obesity in delayed puberty group were significantly lower than that in on-time and early puberty group. This result was consisted with J. Ribeiro’s research [6] but contrary to Wang’s [4] and Fang Qi’s [28]. These disparities could be due to differences in age structure between the studies or the use of variable criteria for judging puberty timing. In addition, for different social and cultural adolescents, eating habits and nutritional status are different, which may also affect the growth and development of boys [31].

Bao et al. [32] reviewed various recent studies that examined the relationship between overweight/obesity, body compositions and puberty events in females and concluded that higher BMI and body fat content before or during puberty can accelerate the onset of puberty. Remer et al. [33] found that increased body fat may be important for the secretion of androgens in girls. Blank et al. [34] believe that the high concentration of androgens in obese girls may promote increased secretion of GnRH, resulting in early puberty. A study by Brill in rodents found that high fat diets lead to rat vaginal opening in advance and that blocking the androgen receptor can reverse this phenomenon [35]. These observations may explain the effect of body fat on puberty that we observed in this study, as we also found in girls that FM in delayed puberty group was significantly lower than that in on-time puberty group or early puberty group. In addition, we also found that the FFM in the delayed puberty group was significantly lower than that in the early puberty group and the on-time puberty group, while BD in the delayed puberty group was significantly higher than that in the early puberty group and the on-time puberty group. These indicated that girls who have low body mass and weight, high BD, could have a delayed puberty. But the FM, FFM, and BD of boys were not significantly related to their timing of puberty. There was no significant difference in body fat and body weight between boys and girls in childhood, but with age, especially after puberty, %BF of boys and girls showed significant difference [36]. Under the influence of sex hormone, the muscle and bone of boys developed rapidly, lead to FFM increased significantly and %BF of boys decreased significantly, while the %BF of girls increased significantly. What’s more, the amount of exercise of boys was usually higher than that of girls, and exercise can increase lean body mass and decrease body fat. Data showed that %BF of 18 years old Chinese boys (17%) was significantly lower than that of Chinese girls (27.8%) [1]. In the whole process of growth and development, the level of serum leptin has great changes. The level of leptin regulated by many factors in the body, in which, the content of adipose tissue is a major factor, the higher fat content, the higher the level of leptin, and leptin have direct or indirect effects on puberty and reproductive regulation [37], which regulates the release of luteinizing hormone releasing hormone (LHRH) in adults, thus directly influences the ovaries and plays an important role in initiation and progression of puberty.

Besides, change of body fat composition and pubertal stages involve extremely complicated genetic and other endocrine processes, and that a final conclusion about the causality between obesity and pubertal development has not been reached in either domestic or international research. The association between puberty onset and development and body fat compositions presented in our survey and others serves as an alert to the importance of considering precocious puberty when looking at high %BF. Ultimately, it is important to remember that these variables are not acting in a vacuum and that other factors should also be considered. Considering socioeconomic status and other potential mediators of puberty may help us to understand the short- and long-term consequences of puberty development with respect to body fat composition and aid in establishing a causal link between them.

### Study strengths and weaknesses

Earlier studies have linked early puberty to higher body fat, at least in girls. However, in most large-scale epidemiologic studies, fat mass has not been directly measured. Usually, BMI is used as a surrogate for body fat. Studies found that no matter for white or black girls, the mean BMI z scores of girls with breast development were significantly greater than for age-matched girls who were prepubertal [38]. There were positive relations between increasing stages of breast development and BMI, waist circumference, fat mass and %BF [39]. However the breast development was assessed by inspection and not palpation, which can cause confusion in overweight girls, in whom fat can mimic breast tissue. As for boys, the relation between body fat and puberty has been controversial. Wang even pointed to the possibility that obesity may actually result in later rather than earlier puberty in boys [4]. In this study, we used the breast in girls and testicular volume in boys as the indicator of pubertal timing, and analysed the relation with fat level, which was classified by BMI, and body compositions respectively. We found that the relations were difference. In boys, the rates of overweight and obesity in early puberty group were significant higher than on-time and delayed puberty group. While the relations between pubertal timing and indicators of body composition didn’t find. In girls, early puberty was positively correlated with obesity, but the body compositions in early puberty had no statistical difference compared with on-time puberty. These indicate that BMI as a surrogate for body fat can chang the real relation between body fat and puberty. Besides, we found that the development of boys’ testicles may not be related to body compositions, but girls’ breast development is not only related to FM, but also to FFM. Girl with lighter weight and higher BD could has later breast development.

Some limitations of the present study still should be noted. First, this is a cross-sectional survey, so causal relationships between variables of interest cannot be assessed. Furthermore, the study populations were only based on two schools in one district of Chongqing. Cohort studies with larger sample size and covering wider age range are still needed.

## Conclusion

In summary, obesity and overweight are negatively correlated with delayed puberty in girls. Body compositions, such as %BF, FM and FFM are negatively correlated with delayed puberty and BD are positively correlated with delayed puberty in girls. But the relation between obesity or body compositions with timing of puberty was not found in boys. Prospective cohort studies are needed to determine the causality of timing of puberty and obesity and also whether the association between body compositions and timing of puberty merit further attention.

## Abbreviations

%BF: Percentage of body fat
BD: Body Density
FFM: Fat-free mass
FM: Fat mass
SST: Sum of skinfold thickness
VOT: Volume of testis
